# Evaluating the Effects of Holidays on Road Crash Injuries in the United Kingdom

**DOI:** 10.3390/ijerph18010280

**Published:** 2021-01-01

**Authors:** Bayu Satria Wiratama, Ping-Ling Chen, Liang-Hao Chen, Wafaa Saleh, Shang-Ku Chen, Hui-Tsai Chen, Hui-An Lin, Chih-Wei Pai

**Affiliations:** 1 Graduate Institute of Injury Prevention and Control, College of Public Health, Taipei Medical University, Taipei 110, Taiwan; bayu.satria@ugm.ac.id (B.S.W.); plchen@tmu.edu.tw (P.-L.C.); konotane.chen99@gmail.com (L.-H.C.); D513107002@tmu.edu.tw (S.-K.C.); Henry0529@gmail.com (H.-T.C.); Sevenoking219@gmail.com (H.-A.L.); 2 Department of Biostatistics, Epidemiology, and Population Health, Faculty of Medicine, Public Health and Nursing, Universitas Gadjah Mada, Yogyakarta City 55281, Indonesia; 3 Department of Emergency Medicine, WanFang Hospital, Taipei Medical University, Taipei 116, Taiwan; 4 Transport Research Institute, Edinburgh Napier University, Scotland EH11 4DY, UK; w.saleh@napier.ac.uk; 5 Department of Engineering, Princess Nora Bint Abdul Rahman University, Riyadh 11564, Saudi Arabia; 6 Department of Emergency Medicine, Shuang Ho Hospital, Taipei Medical University, Taipei 235, Taiwan; 7 Department of Emergency Medicine, Keelung Hospital, Ministry of Health and Welfare, Keelung 201, Taiwan; 8 Department of Emergency Medicine, Taipei Medical University Hospital, Taipei 110, Taiwan

**Keywords:** public holiday, killed or seriously injured, fatal injury

## Abstract

Background: Research suggests that drivers tend to engage in risk-taking behaviours on public holidays. Studies that examined the association between holidays (or other special days) and fatal injuries are inconsistent. This study used UK STATS19 data to investigate the associations of nine public holidays on road crash casualties. Methods: This retrospective study assessed UK STATS19 crash data for 1990–2017. All casualties from two vehicle crashes were initially considered; subsequently, casualties with missing data were excluded. Multiple logistic regression was estimated to explore the associations of potential risk factors with the likelihood of killed or seriously injured (KSI) casualties and to calculate adjusted odds ratios (AORs). Results: In total, 3,751,998 casualties from traffic accidents in the United Kingdom during 1990–2017 were included in the final data set; among these, 410,299 (10.9%) were KSI casualties, and 3,341,699 (89.1%) were slight injuries. Crashes on public holidays were 16% (AOR = 1.16; 95% confidence interval [CI] = 1.13–1.19) more likely to involve KSI casualties than were crashes on non-holidays. With other factors controlled for, crashes during the Queen’s 2002 Golden Jubilee and on New Year’s Day were 48% (AOR = 1.48; 95% CI = 1.06–2.07) and 36% (AOR = 1.36; 1.26–1.48) more likely to lead to KSIs, respectively. Conclusions: The proportion of crashes resulting in KSI casualties on public holidays was higher than that on non-holidays. Furthermore, crashes during the Queen’s 2002 Golden Jubilee had the highest risk of KSI casualties followed by New Year’s Day.

## 1. Introduction

The World Health Organization estimated that 1.4 million deaths are caused by traffic injuries annually [1]. In 2018, in the United Kingdom, 160,378 casualties, including 1782 fatalities, from road traffic crashes were reported to the police; moreover, the road users who resulted in being the highest proportion of traffic fatalities were car occupants (44%), followed by pedestrians (25%) and motorcyclists (20%) [2]. Major risk factors for traffic fatalities include sleep deprivation, distracted driving, and fatigue [3,4,5,6].

In the United Kingdom, public holidays include religious and bank holidays. On bank holidays, banks, government offices, and most businesses are closed. Religious holidays in the United Kingdom include Easter Monday, Halloween, Christmas, and Boxing Day. Several studies have examined the effects of holidays on crash risks. Philip et al. [7] investigated sleepiness among automobile drivers and concluded that approximately 50% of drivers on their summer vacations had a significant degree of sleep deprivation prior to departure. By contrast, Zhang et al. [8] reported no significant association between holidays and fatigue-related crashes. Bloch et al. [9] reported an increase in drunk driving during the winter holiday season in California. Studies using traffic crash data in Canada have concluded that fatal injuries and the non-use of restraints (i.e., seat belts) are more common on statutory holidays [10].

Studies on intoxicated driving on holidays tend to be relatively scant. Foster et al. [11] reported an increase in alcohol-related crashes in Switzerland from holiday eves until 3 days after those holidays. Mäkelä et al. [12] reported similar statistics in Finland; Christmas, the May Day Festival, and the Midsummer Festival were associated with significantly higher risks of alcohol-related traffic fatalities.

Studies that examined the association between holidays (or other special days) and fatal injuries have yielded inconsistent results. For example, Farmer and Williams [13] examined Fatality Analysis Reporting System (FARS) crash data from the United States and reported a disproportionately high number of fatal injuries around the time of Independence Day, Christmas, New Year’s Day, and Labor Day. Other US studies have revealed similar findings. For example, studies have discovered increases in fatal vehicle crashes on Super Bowl Sunday, presidential election day, and Halloween [14,15,16]. Using FARS crash data, Zhang and Aronow [17] reported a conflicting result; presidential election days were not associated with fatal road crashes. Evidence suggests that in Malaysia, conspicuity-related motorcycle crashes are more common during Ramadan but not during the balik kampung season, when people usually return to their hometowns [18]. After analysing nearly 8000 vehicles involved in crashes, Zhang et al. [19] identified an association between public holidays and fatal injuries involving overloaded vehicles.

Traffic violations, intoxicated driving, and seat belt non-use were determined to be significant predictors of severe injuries resulting from vehicle crashes during public holidays in Canada [20]. Comparing public holidays with normal weekends, Anowar et al. [10] discovered that in holiday crashes, driving without a seat belt was more common, whereas speeding and drunk driving were less common. By contrast, Zhang et al. [21] reported an elevated incidence of drunk driving in China during public holidays.

When reviewed together, previous results on the relationship between holidays and fatal crashes are inconsistent [14,16,17,18,19,22,23]. Most studies have analysed one specific holiday or holidays generally, but comparisons of different holidays are rare. Using UK STATS19 data for 1990–2017, we investigated the effects of nine public holidays on traffic crash severity in the United Kingdom.

## 2. Materials and Methods

### 2.1. Study Design and Setting

This retrospective study analyzed secondary data on UK traffic dataset using UK STATS19 crash data for 1990–2017. The UK STATS19 is a national traffic dataset which includes data for every crash resulting in personal injuries reported to the UK police within 30 days [24]. STATS19 collected data on all personal injury road crashes on public highways involving at least one vehicle, reported to and recorded by the police. Drivers were not obliged to report personal injury and only accident involving other injured drivers/pedestrian was included as an obligation to report to the police according to the UK road traffic act (1988) section 170 [25,26]. This database was established in 1949 and had been periodically reviewed and modernized. The UK STATS19 has records regarding crash, vehicle, and casualty characteristics. The crash file records data on crash time, date, weather, road and light conditions. Next, the vehicle file contains data on vehicle and driver details. Lastly is the casualty file which contains data about details for each casualty. The UK STATS19 data are owned by the Department for Transport, the United Kingdom [24]. This dataset is available for public in the website of Department of Transport [24]. This study was approved by Taipei Medical University (TMU) Joint Institutional Review Board (N202011030) [27].

### 2.2. Casualties

**Figure 1:**
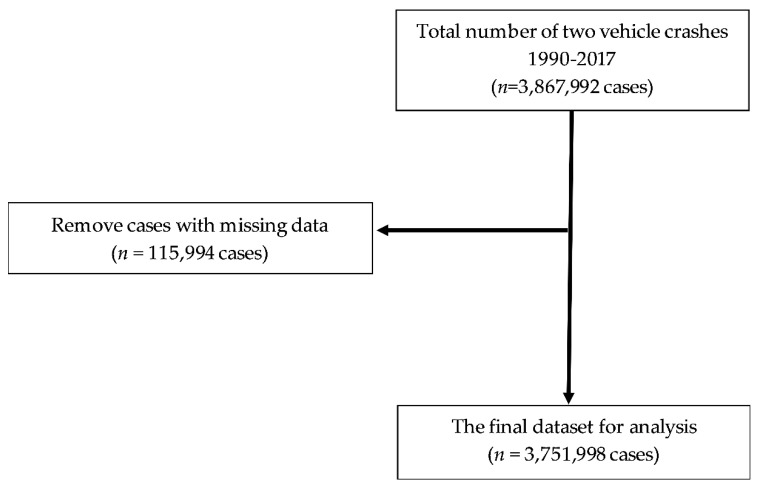
Flowchart of data sampling (*n* = number of cases).

This study focused on two vehicle crashes involving cyclists, motorcyclists, and motorists using the UK STATS19 data [24]. Figure 1 describe a flowchart of data selection for this study. We excluded casualties with missing data for sex, age, speed limit, collision time, or vehicle type. A complete case analysis approach was used in this study as proposed by Kang [28]. There is no difference between cases with and without missing data (*p* > 0.05). A total of 3,751,998 casualties were included in the final dataset.

### 2.3. Outcome and Variable Definitions

This study defined casualties as either killed or seriously injured (KSI) or slight injuries. Slight injuries included mild injuries such as sprains (including whiplash), bruises, and cuts as well as mild shock requiring roadside attention. Injuries not requiring medical treatment were also classified as slight injuries. This study collected data on crash and driver’s/rider’s characteristics and identified the specific public holiday (if any) on which the crash occurred. The driver characteristics analysed were sex (male or female) and age (≤17, 18–40, 41–64, or ≥65 y). We collected data on vehicle type for both the driver and the crash partner. Vehicle type was bicycles, motorcycles, automobiles, and large vehicles. Lighting conditions were categorised as either night (sunset until sunrise) or day (sunrise until sunset). Weather was categorised as either fine or adverse. Road type was determined from speed limit data; roads with speed limits of <30 m per hour were considered urban, and those with speed limits of ≥30 m per hour were considered rural. We classified four crash types: rear-end crash, sideswipe, head-on crash, and other type of crash [29,30,31].

Bank holidays (public holidays) are national public holidays in the United Kingdom. The term is commonly used to include other public holidays such as Good Friday and Christmas. Nine UK holidays were considered in this study: New Year’s Day, Good Friday, Easter Monday, summer bank holidays, spring bank holidays, Christmas, Boxing Day, the Queen’s 2002 Golden Jubilee, and the Queen’s 2012 Diamond Jubilee.

### 2.4. Statistical Analysis

We first compared the distribution of casualties across age, sex, lighting conditions, weather conditions, road type, vehicle type, holiday, and injury severity (KSI or slight injury). A chi-square test was used to estimate homogeneity between KSI groups. Since all of our data were in categorical form then we decided not to conduct any normality assumption test. Simple logistic regression was used to examine the associations between KSI casualties and risk factors. Following previous research [32,33], we used a *p* value of <0.2 to identify independent variables, and they were then included in a multiple logistic regression model to explore the associations of potential risk factors with KSI casualties and to calculate adjusted odds ratios (AORs). Cramer’s *V* was used to assess multicollinearity according to previous research [29,32,34]. An alpha of 0.05 was used, yielding a confidence level of 95%. Complete case analysis was used in this study. Missing data were considered to be at least missing at random and were, therefore, excluded from the analysis [28].

## 3. Results

**Figure A1:**
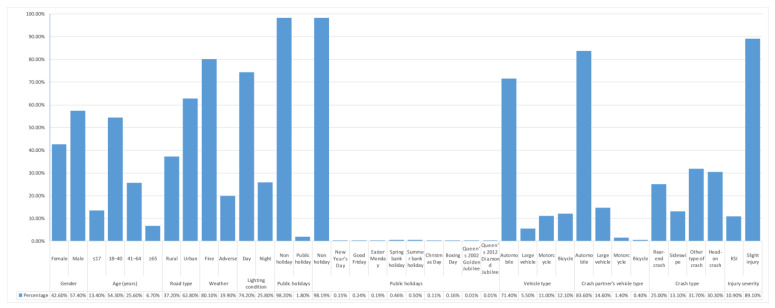
Distribution of casualties’ characteristics from the UK STATS19 data [24].

Table 1 and Figure A1 shows the distribution of casualties for each variable. During 1990–2017, there were 3,751,998 casualties included in this study, of which 410,299 (10.9%) were considered KSIs and 3,341,699 (89.1%) were considered slight injuries. We found 57.4% of casualties were male. The majority of casualties in this study were in age group of 18–40 years (54.3%). Automobiles (71.4%) were the vehicle type most involved in road crashes. The number of casualties in urban areas (62.8%) outnumbers those in rural areas (37.2%). The number of casualties were more prevalent on Summer bank holiday (0.50%) and Spring bank holiday (0.46%) compared with other public holidays.

**Figure A2:**
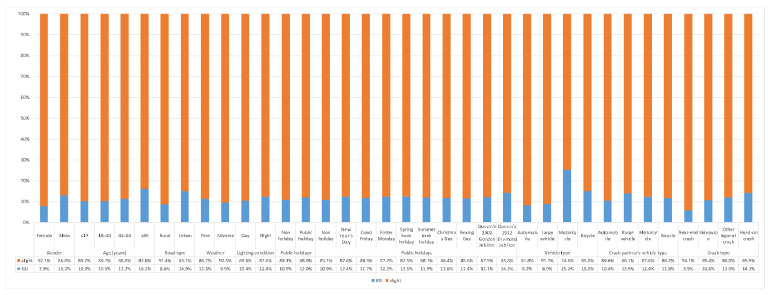
Distribution of injury severity for each independent variables in the UK STATS19 data [24].

Table 2 and Figure A2 presents the distribution of injury severity for each independent variable. Male occupants (13.2%) were more likely to sustain KSIs than female ones (7.9%). The proportion of KSIs among those 65 years or older (16.2%) was higher than among other age groups. The risk of KSIs was higher on rural roads (14.9%) than on urban roads (8.6%). The proportion of KSIs (12.0%) was higher on public holidays than on normal days (10.9%). Among all public holidays, the Queen’s 2012 Diamond Jubilee (14.2%) had the highest proportion of crashes resulting in KSIs compared with the other public holidays. Motorcycle riders (25.2%) had the highest percentage of KSIs, followed by bicycle riders (15.0%), large vehicle drivers (8.9%), and automobile drivers (8.2%). Among crash partner vehicle type, vehicle drivers involved in crashes with large vehicles (13.9%) had the highest percentage of KSIs compared with other vehicle types. The result of chi-square tests showed that there is heterogeneity between KSI group.

**Figure A3:**
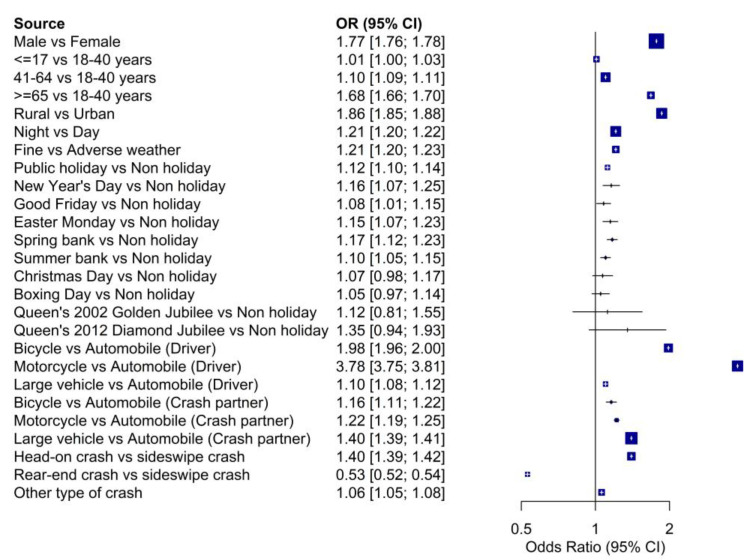
Forest plot of odds ratio from simple logistic regression analysis for each independent variables using the UK STATS19 data [24].

Table 3 and Figure A3 shows the simple logistic regression results for various risk factors of KSIs including sex, age, road type, lighting conditions, weather conditions, public holidays, and vehicle type. Drivers involved in crashes during public holidays had 12% (OR = 1.12; 95% confidence [CI] = 1.10–1.14) higher risks of sustaining KSIs compared to those involved in crashes on non-holidays. Furthermore, both Spring bank holiday (OR = 1.17; 95% CI = 1.11–1.22) and New Year’s day (OR = 1.16; 95% CI = 1.07–1.25) had the highest risks of KSIs among all UK public holidays. Motorcycle riders had approximately 3.78 times higher risks (OR = 3.78; 95% CI =3.75–3.81) to sustain KSIs compared with automobile drivers. Drivers involved in crashes with large vehicles were 40% (OR = 1.40; 95% CI = 1.39–1.41) more likely to sustain KSIs than drivers involved in crashes with automobiles. Casualties were more likely to sustain KSIs if they were male (OR = 1.77; 95% CI = 1.76–1.78), older than 65 years (OR = 1.68; 95% CI = 1.66–1.70), driving on rural roads (OR = 1.86; 95% CI = 1.84–1.87), driving at night time (OR = 1.21; 95% CI = 1.20–1.22), and driving under fine weather (OR = 1.21; 95% CI = 1.19–1.22).

**Figure A4:**
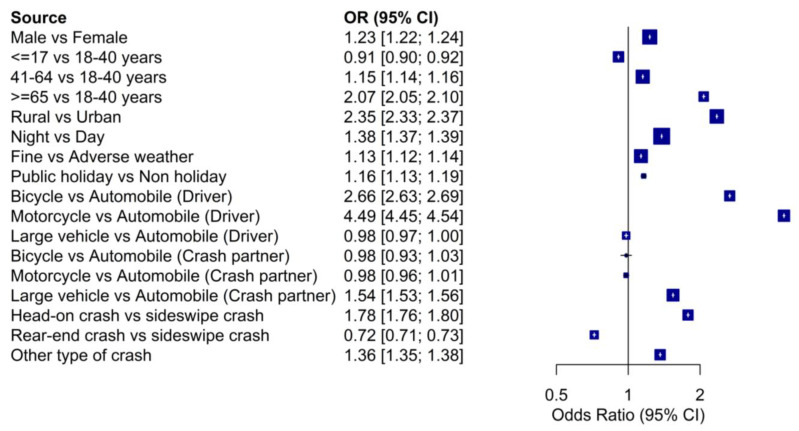
Forest plot of multiple logistic regression result for each independent variables using the UK STATS19 data [24].

Table 4 and Figure A4 lists the results of the multiple logistic regression model for KSIs. Drivers/riders involved in crashes during public holidays were 16% (adjusted odds ratio (AOR) = 1.16; 95% CI = 1.13–1.19) more likely to sustain KSIs than were those on non-holidays. Compared with automobiles, those riding motorcycles had an increased probability of sustaining KSIs by 349% (AOR = 4.49; 95% CI = 4.44–4.53). Among all age groups, drivers/riders aged 65 or above had the highest risks for KSIs (AOR = 2.07; 95% CI = 2.05–2.10). Drivers involved in crashes with large vehicle had an increased probability of sustaining KSIs by 54% (AOR = 1.54; 95% CI = 1.53–1.55) than drivers involved in crashes with automobiles. Other risk factors of KSIs were male (AOR = 1.23; 95% CI = 1.22–1.24), night time (AOR = 1.38; 95% CI = 1.37–1.39), fine weather (AOR = 1.13; 95% CI = 1.12–1.14), rural roadways (AOR = 2.35; 95% CI = 2.33–2.37), and riding bicycles (AOR = 2.66; 95% CI = 2.63–2.69). There was no multicollinearity between risk factors in this study.

**Figure A5:**
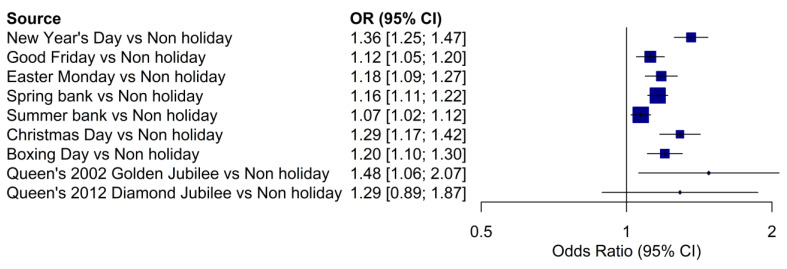
Forest plot of odds ratio from multiple logistic regression by different type of holidays using the UK STATS19 data [24].

Table 5 and Figure A5 reports different types of public holidays using multiple logistic regression models. The Queen’s 2002 Golden Jubilee and New Year’s Day had the highest AORs of KSIs among public holidays in the United Kingdom. Drivers/riders involved in crashes during Queen’s 2002 Golden Jubilee and New Year’s Day were 48% (AOR = 1.48; 95% CI = 1.06–2.07) and 36% (AOR = 1.36; 1.26–1.48) more likely to sustain KSIs after controlling for other risk factors. Compared with non-holidays, the other public holidays were also significantly associated with higher risks of KSIs except for the Queen’s 2012 Diamond Jubilee holiday.

## 4. Discussion

One of the most crucial findings in this study is that public holidays were associated with higher risks of KSIs after controlling for other risk factors. Our study corroborates with current literature that there was a disproportionate number of fatal injuries on holidays [13,14,15,16,18,19]. This finding can attribute to higher proportions of drivers engaging in risk-taking behaviours, such as seat belt non-use and drunk driving, on public holidays [10,21]. A Finnish study also lends support to our finding: Mäkelä et al. [12] concluded that Christmas, May Day Festival, and the Midsummer Festival were significantly associated with higher risks of KSIs.

The Queen’s Golden Jubilee was a national public holiday in 2002 that celebrated the 50th anniversary of Queen Elizabeth’s accession, and it consisted of several festivals. We demonstrated that traffic casualties, including those involving motorcycles, during the Queen’s Golden Jubilee were more likely to be KSIs. The literature has suggested that drunk driving and riding without a helmet are more common on festival days such as Chinese New Year and the Songkran Festival [21,35]. In Switzerland, a past study reported that there is an increase in alcohol-related crashes from holiday eves to 3 days after those holidays [11]. Our conjecture should be supported by additional data related to alcohol and helmet use. Other factors may include distracted driving during festivals and rerouting to unfamiliar roads, both of which may increase the likelihood of traffic violations [36].

In accordance with previous literature [37,38,39,40], this study concluded that males had an increased risk of KSIs than females. This finding is likely attributable to the higher proportion of men engaging in risk-taking behaviours such as driving under the influence of alcohol, speeding, driving without a seat belt and riding without a helmet [40,41]. Moreover, professional drivers were more likely to have fatigue-related crashes which could aggravate injury severity, and professional drivers are predominantly male [8,42].

This study revealed results consistent with those of previous studies; road crashes on rural roadways were more likely to result in KSIs than were those on urban roadways. This results may be due to the higher incidence of speeding and drunk driving in rural areas than urban areas [21,43]; both behaviours were associated with fatal injuries [32,44,45,46,47,48].

Corroborating the findings of previous studies, we discovered that the risks of KSIs were higher at night time than during day [40,48,49]. Motion-based perception is impaired at night, increasing the risk of a crash [50,51]. Furthermore, visibility is limited, even with high beam light,; thus, the driver’s time to react is reduced, especially at high speeds [52].

We also revealed vehicle type as a significant predictor of KSIs, which is a result similar to those of other studies [53,54,55]. This result is attributed to the different protective measures across vehicle types. Motorcyclists and cyclists are less protected than drivers of automobiles and heavy vehicles [53].

Our finding that a head-on crash is an important predictor of fatal injuries confirms the results of the existing literature [29,30,31,32,56,57]. This finding may be due to the fact that speeding is more likely to occur during a head-on crash [58,59]. Due to the high impact force that exceeds the human body threshold, speeding is highly correlated with fatal injuries [60]. In addition, drivers involved in a head-on crash were more likely to suffer head, chest and neck injuries [61,62,63,64].

Finally, older drivers were more likely to sustain KSIs, which is consistent with the results in the literature [65]. Older drivers experience cognitive decline. Lundberg et al. [66] discovered that older drivers had lower performance on cognitive function than younger drivers. Moreover, poor vision, slow processing speeds, decline in physical ability, and the side effects of medications for chronic diseases may increase older driver’s risk of crashing [67,68,69,70].

This study has several limitations. First, data were limited for some independent variables, such as vehicle speed, traffic volume, use of communication devices, emergency medical team response time, and distance to the nearest trauma-level hospital, which may crucially affect injury severity. Second, this study used a UK STATS19 database in which detailed injury outcomes were not available. A combined dataset with hospital based data may provide greater detail regarding injury outcomes, such as the injured regions of the body and duration of treatment. Lastly, we only included two vehicle crashes. Our result could not be applied for multi vehicle crashes involving more than two vehicles.

## 5. Conclusions

Our main finding is the association of public holidays in the UK with KSIs. Furthermore, among all holidays, road crashes that occurred during the Queen’s 2002 Golden Jubilee had the highest risk of KSIs followed by New Year’s Day. This finding may be due to higher proportions of drivers engaging in risk-taking behaviours, such as seat belt nonuse and drunk driving, on public holidays. Further research is needed to obtain more detailed results about specific risk factors of KSIs during public holidays, such as alcohol consumption, speeding and helmet use.

## Figures and Tables

**Table 1:** Characteristics of casualties from 1990–2017 (Data based on UK STATS19 1990–2017) [24].

Characteristics	Number of Casualties (*n*)	Percentage (%)
Sex		
Female	1,598,979	42.6
Male	2,153,019	57.4
Driver’s age		
≤17 years	504,442	13.4
18–40 years	2,037,233	54.3
41–64 years	959,904	25.6
≥65 years	250,419	6.7
Road type		
Urban	1,397,266	37.2
Rural	2,354,732	62.8
Weather		
Fine	3,006,380	80.1
Adverse	745,618	19.9
Lighting condition		
Day	2,782,762	74.2
Night	969,236	25.8
Public holiday		
Non holiday	3,683,987	98.2
Public holiday	68,011	1.8
Public holiday		
Non holiday	3,683,987	98.19
New Year’s Day	5529	0.15
Good Friday	8995	0.24
Easter Monday	7139	0.19
Spring bank holiday	17,114	0.46
Summer bank holiday	18,760	0.50
Christmas Day	4009	0.11
Boxing Day	5870	0.16
Queen’s 2002 Golden Jubilee	348	0.01
Queen’s 2012 Diamond Jubilee	247	0.01
Vehicle type		
Automobile	2,677,961	71.4
Large vehicle	206,965	5.5
Motorcycle	413,863	11.0
Bicycle	453,209	12.1
Crash partner vehicle type		
Automobile	3,135,481	83.6
Large vehicle	546,738	14.6
Motorcycle	52,902	1.4
Bicycle	16,877	0.4
Crash type		
Rear-end crash	936,691	25.0
Sideswipe	491,465	13.1
Other type of crash	1,188,627	31.7
Head-on crash	1,135,215	30.3
Injury severity		
KSI	410,299	10.9
Slight injury	3,341,699	89.1
Total	3,751,998	100.0

**Table 2:** Distribution of injury severity by risk factor during 1990–2017 (Data based on UK STATS19 1990–2017) [24].

Characteristics	Killed or Seriously Injured (KSI) *n* (%)	Slight Injury *n* (%)	*p*-Value
Sex			<0.001
Female	126,295 (7.9%)	1,472,684 (92.1%)	
Male	284,004 (13.2%)	1,869,015 (86.8%)	
Driver’s age			<0.001
≤17 years	52,137 (10.3%)	452,305 (89.7%)	
18–40 years	209,736 (10.3%)	1,827,497 (89.7%)	
41–64 years	107,955 (11.2%)	851,949 (88.8%)	
≥65 years	40,471 (16.2%)	209,948 (83.8%)	
Road type			<0.001
Urban	202,542 (8.6%)	2,152,190 (91.4%)	
Rural	207,757 (14.9%)	1,189,509 (85.1%)	
Weather			<0.001
Fine	339,131 (11.3%)	2,667,249 (88.7%)	
Adverse	71,168 (9.5%)	674,450 (90.5%)	
Lighting condition			<0.001
Day	290,460 (10.4%)	2,492,302 (89.6%)	
Night	119,839 (12.4%)	849,397 (87.6%)	
Public holiday			<0.001
Non holiday	402,109 (10.9%)	3,281,878 (89.1%)	
Public holiday	8190 (12.0%)	59,821 (88.0%)	
Public holiday			<0.001
Non holiday	402,109 (10.9%)	3,281,878 (89.1%)	
New Year’s Day	686 (12.4%)	4843 (87.6%)	
Good Friday	1048 (11.7%)	7947 (88.3%)	
Easter Monday	881 (12.3%)	6258 (87.7%)	
Spring bank holiday	2137 (12.5%)	14,977 (87.5%)	
Summer bank holiday	2228 (11.9%)	16,532 (88.1%)	
Christmas Day	466 (11.6%)	3543 (88.4%)	
Boxing Day	667 (11.4%)	5203 (88.6%)	
Queen’s 2002 Golden Jubilee	42 (12.1%)	306 (87.9%)	
Queen’s 2012 Diamond Jubilee	35 (14.2%)	212 (85.8%)	
Vehicle type			<0.001
Automobile	219,321 (8.2%)	2,458,640 (91.8%)	
Large vehicle	18,508 (8.9%)	188,457 (91.1%)	
Motorcycle	104,366 (25.2%)	309,497 (74.8%)	
Bicycle	68,104 (15.0%)	385,105 (85.0%)	
Crash partner vehicle type			<0.001
Automobile	325,552 (10.4%)	2,809,929 (89.6%)	
Large vehicle	76,218 (13.9%)	470,520 (86.1%)	
Motorcycle	6534 (12.4%)	46,368 (87.6%)	
Bicycle	1995 (11.8%)	14,882 (88.2%)	
Crash type			<0.001
Rear-end crash	55,210 (5.9%)	881,841 (94.1%)	
Sideswipe	51,929 (10.6%)	439,536 (89.4%)	
Other type of crash	143,217 (12.0%)	1,045,410 (88.0%)	
Head-on crash	159,943 (14.1%)	975,272 (89.1%)	

**Table 3:** Simple logistic regression results of factors associated with KSI casualties (data based on UK STATS19 1990–2017) [24].

Variable	β	OR (95% CI)	*p*-Value
Sex			
Male	0.57	1.77 (1.76–1.78)	<0.001
Female (Ref.)	-	1	
Age (y)			
≤17	0.04	1.01 (0.99–1.02)	0.020
18–40 (Ref.)	-	1	
41–64	0.10	1.10 (1.09–1.11)	<0.001
≥65	0.52	1.68 (1.66–1.70)	<0.001
Road type			
Rural	0.62	1.86 (1.84–1.87)	<0.001
Urban (Ref.)		1	
Lighting condition			
Night	0.19	1.21 (1.20–1.22)	<0.001
Day (Ref.)		1	
Weather			
Fine	0.19	1.21 (1.19–1.22)	<0.001
Adverse (Ref.)	-	1	
Public holiday			
Public holiday	0.11	1.12 (1.10–1.14)	<0.001
Non holiday (Ref.)	-	1	
Public holiday			
New Year’s Day	0.15	1.16 (1.07–1.25)	<0.001
Good Friday	0.07	1.08 (1.01–1.15)	0.025
Easter Monday	0.14	1.15 (1.07–1.23)	<0.001
Spring bank holiday	0.15	1.17 (1.11–1.22)	<0.001
Summer bank holiday	0.10	1.10 (1.05–1.15)	<0.001
Christmas Day	0.07	1.07 (0.98–1.18)	0.150
Boxing Day	0.05	1.05 (0.97–1.14)	0.272
Queen’s 2002 Golden Jubilee	0.11	1.12 (0.81–1.55)	0.490
Queen’s 2012 Diamond Jubilee	0.30	1.35 (0.94–1.93)	0.102
Non holiday (Ref.)	-	1	
Vehicle type			
Bicycle	0.68	1.98 (1.96–2.00)	<0.001
Motorcycle	1.33	3.78 (3.75–3.81)	<0.001
Large vehicle	0.10	1.10 (1.08–1.12)	<0.001
Automobile (Ref.)	-	1	
Crash partner vehicle type			
Bicycle	0.15	1.16 (1.10–1.21)	<0.001
Motorcycle	0.20	1.22 (1.19–1.25)	<0.001
Large vehicle	0.34	1.40 (1.39–1.41)	<0.001
Automobile (Ref.)	-	1	
Crash type			
Head-on crash	0.33	1.40 (1.38–1.41)	<0.001
Rear-end crash	−0.64	0.53 (0.52–0.54)	<0.001
Other type of crash	0.15	1.06 (1.05–1.08)	<0.001
Sideswipe crash	-	1	

**Table 4:** Multiple logistic regression results of factors associated with KSIs (data based on UK STATS19 1990–2017) [24].

Variable	β	OR (95% CI)	*p*-Value
Sex			
Male	0.21	1.23 (1.22–1.24)	<0.001
Female (Ref.)	-	1	
Age (y)			
≤17	−0.09	0.91 (0.90–0.92)	<0.001
18–40 (Ref.)	-	1	
41–64	0.14	1.15 (1.14–1.16)	<0.001
≥65	0.73	2.07 (2.05–2.10)	<0.001
Road type			
Rural	0.85	2.35 (2.33–2.37)	<0.001
Urban (Ref.)	-	1	
Lighting condition			
Night	0.32	1.38 (1.37–1.39)	<0.001
Day (Ref.)	-	1	
Weather			
Fine	0.12	1.13 (1.12–1.14)	<0.001
Adverse (Ref.)	-	1	
Public holiday			
Public holiday	0.15	1.16 (1.13–1.19)	<0.001
Non holiday (Ref.)	-	1	
Vehicle type			
Bicycle	0.98	2.66 (2.63–2.69)	<0.001
Motorcycle	1.50	4.49 (4.44–4.53)	<0.001
Large vehicle	−0.02	0.98 (0.96–0.99)	0.012
Automobile (Ref.)	-	1	
Crash partner vehicle type			
Bicycle	−0.02	0.98 (0.93–1.03)	0.330
Motorcycle	−0.07	0.98 (0.96–1.01)	0.216
Large vehicle	0.43	1.54 (1.53–1.56)	<0.001
Automobile (Ref.)	-	1	
Crash type			
Head-on crash	0.58	1.78 (1.76–1.80)	<0.001
Rear-end crash	−0.34	0.72 (0.71–0.73)	<0.001
Other type of crash	0.31	1.36 (1.35–1.38)	<0.001
Sideswipe crash	-	1	
Pseudo R^2^ = 0.116			

**Table 5:** Multiple logistic regression results of factors associated with KSIs by different type of holiday (data based on UK STATS19 1990–2017) [24].

Variable	β	OR (95% CI)	*p*-Value
Public holiday ^a^			
New Year’s Day	0.31	1.36 (1.26–1.48)	<0.001
Good Friday	0.12	1.12 (1.05–1.20)	0.001
Easter Monday	0.16	1.18 (1.09–1.27)	<0.001
Spring bank holiday	0.15	1.16 (1.10–1.21)	<0.001
Summer bank holiday	0.07	1.07 (1.02–1.12)	0.004
Christmas Day	0.25	1.29 (1.17–1.42)	<0.001
Boxing Day	0.18	1.20 (1.10–1.30)	<0.001
Queen’s 2002 Golden Jubilee	0.39	1.48 (1.06–2.07)	0.022
Queen’s 2012 Diamond Jubilee	0.25	1.29 (0.89–1.87)	0.182
Non holiday (Ref.)	-	1	
Pseudo R^2^ = 0.116			

^a^ adjusted for gender, age, weather condition, rural/urban road, lighting condition, vehicle type, crash partner vehicle type, crash type.

## Data Availability

The UK STATS19 data is available for public [24]. The data used for this study is available by request to the authors.
